# Supplementary figures and images for: A C-Terminally Encoded Peptide, MeCEP6, Promotes Nitrate Uptake in Cassava Roots
Source: Plants (Basel). 2025 Apr 21;14(8):1264. doi: 10.3390/plants14081264 (PMC12030203; doi:10.3390/plants14081264)

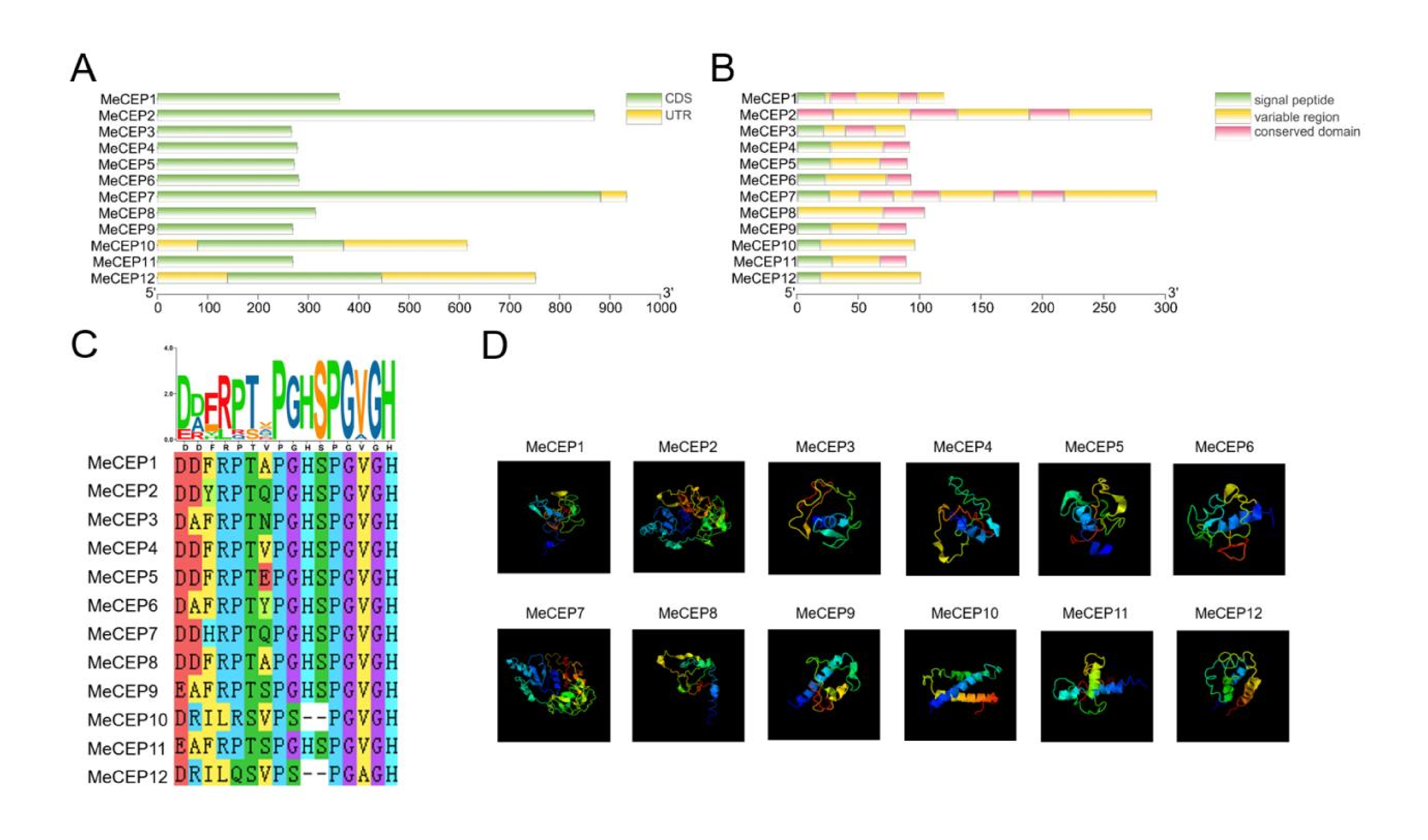

Supplement: Supplementary file 1 [file plants-14-01264-s001.zip › Supplementary Figure S1.tif]

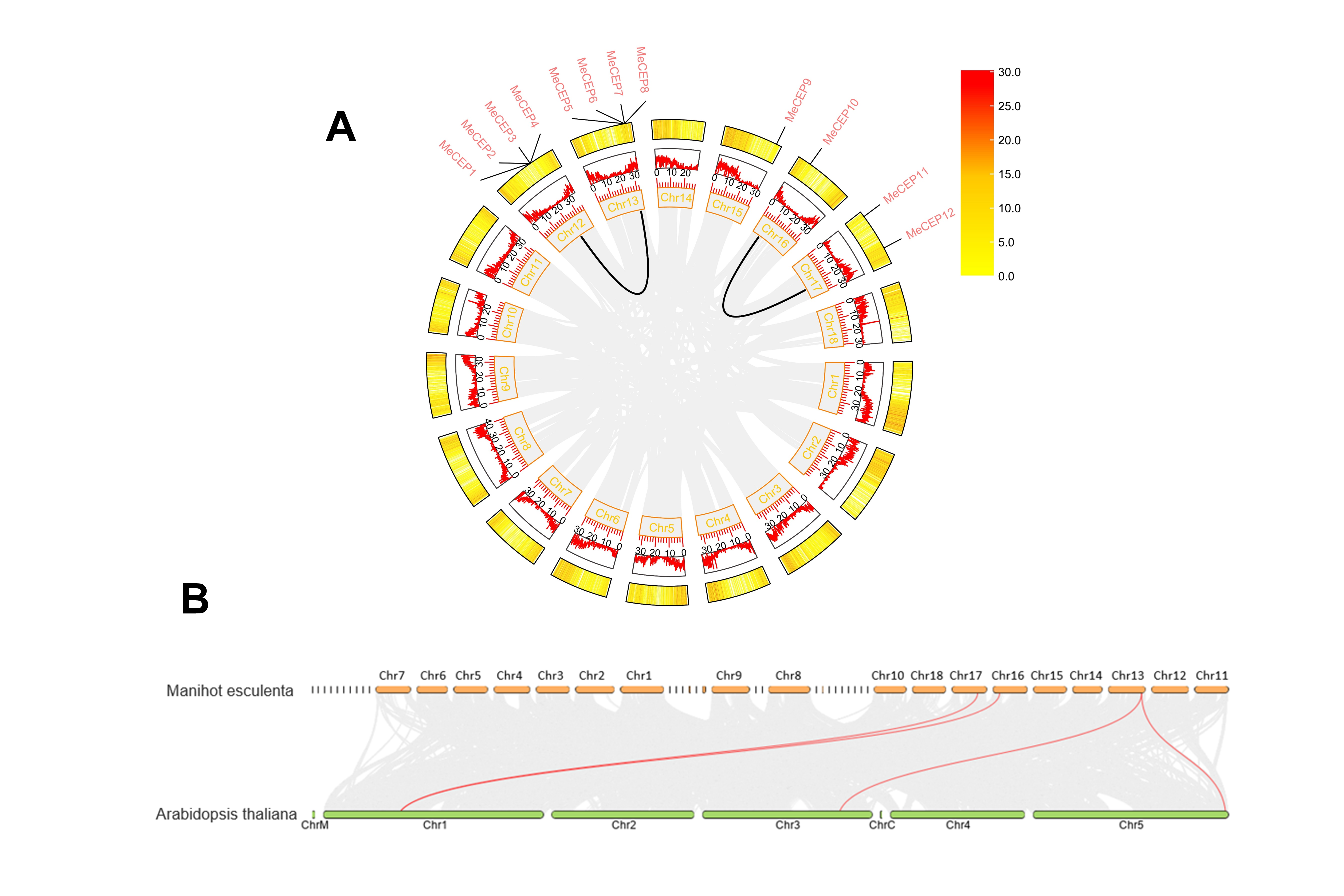

Supplement: Supplementary file 1 [file plants-14-01264-s001.zip › Supplementary Figure S2.tif]

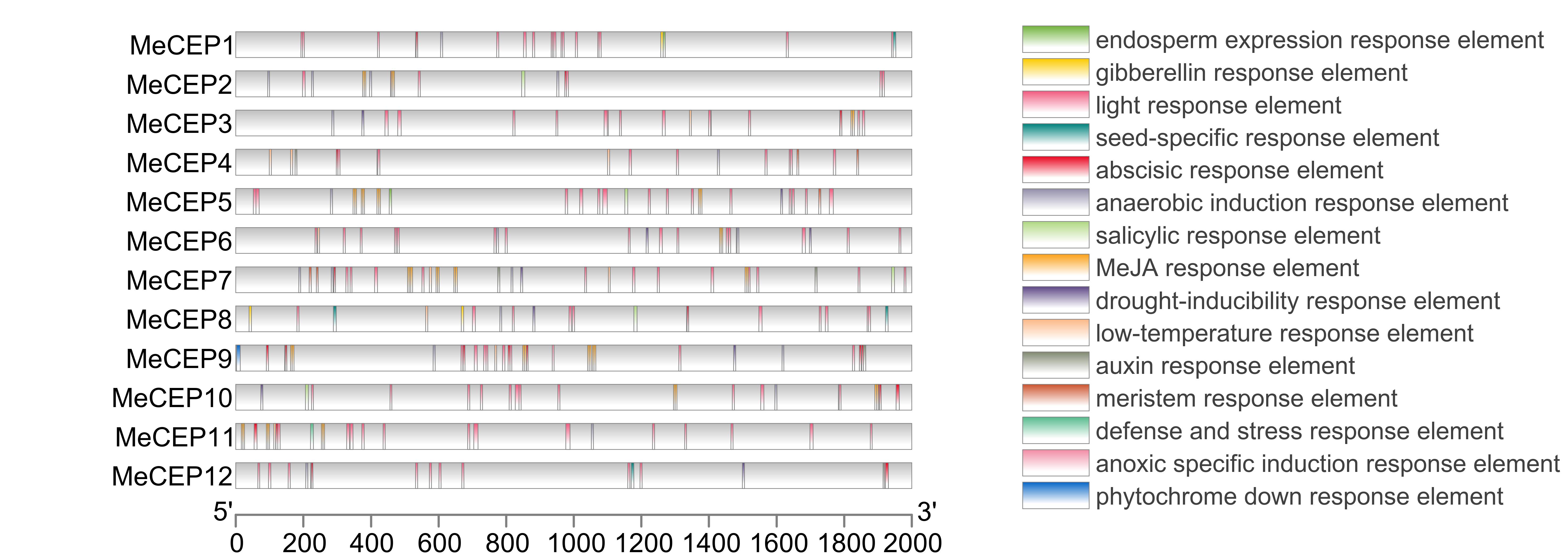

Supplement: Supplementary file 1 [file plants-14-01264-s001.zip › Supplementary Figure S3 .tif]

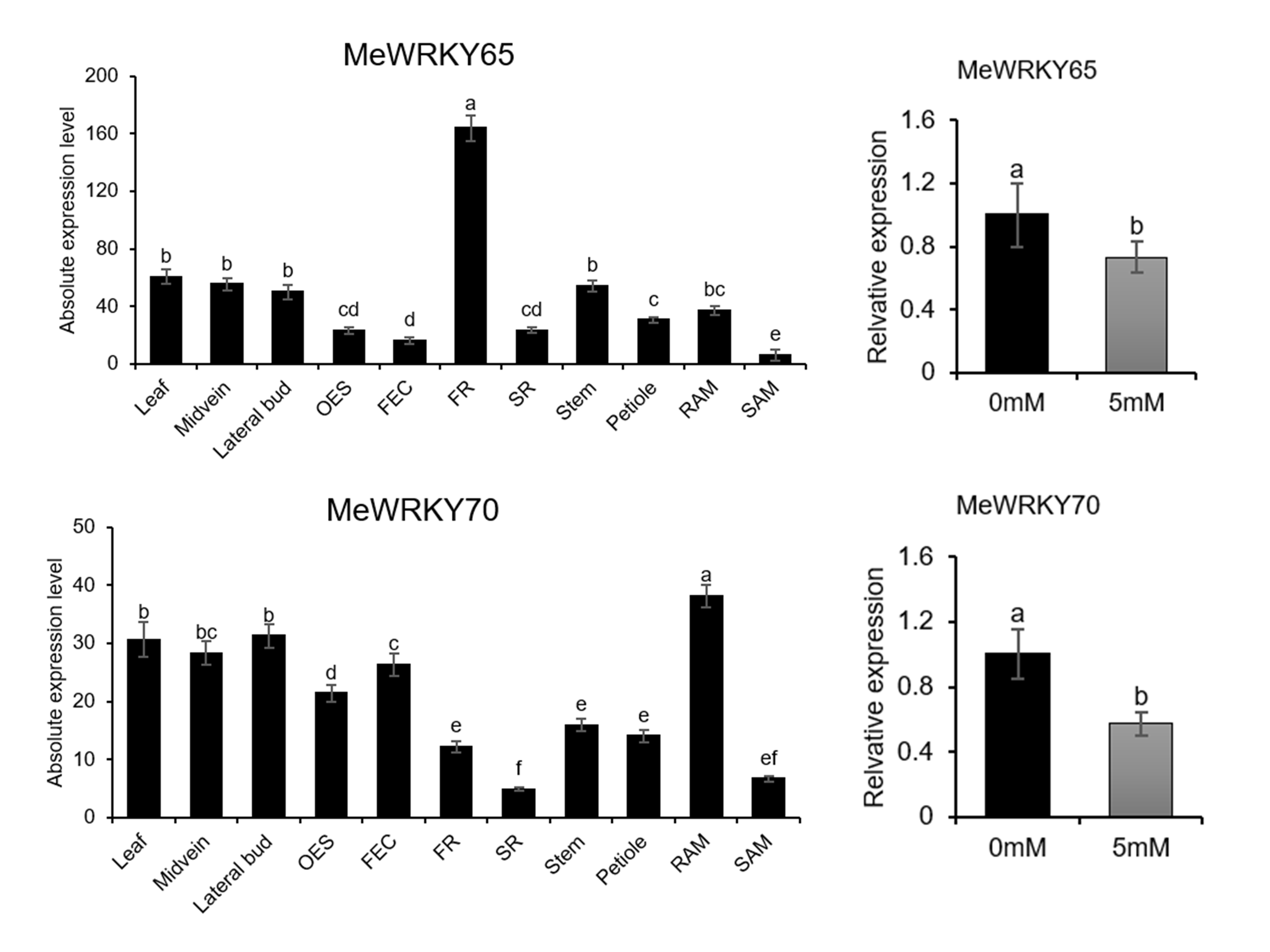

Supplement: Supplementary file 1 [file plants-14-01264-s001.zip › Supplementary Figure S5.tif]

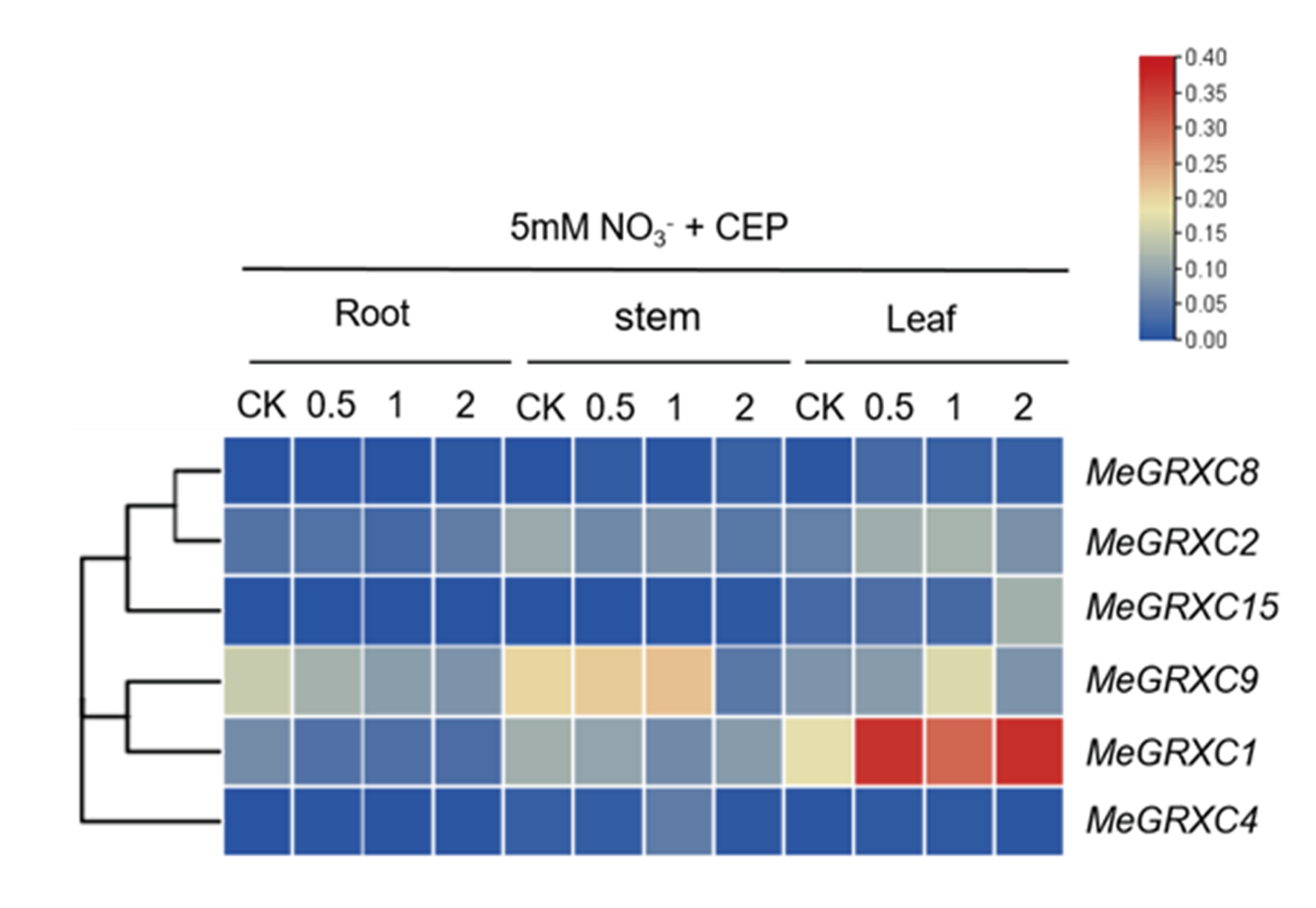

Supplement: Supplementary file 1 [file plants-14-01264-s001.zip › Supplementary Figure S6.tif]

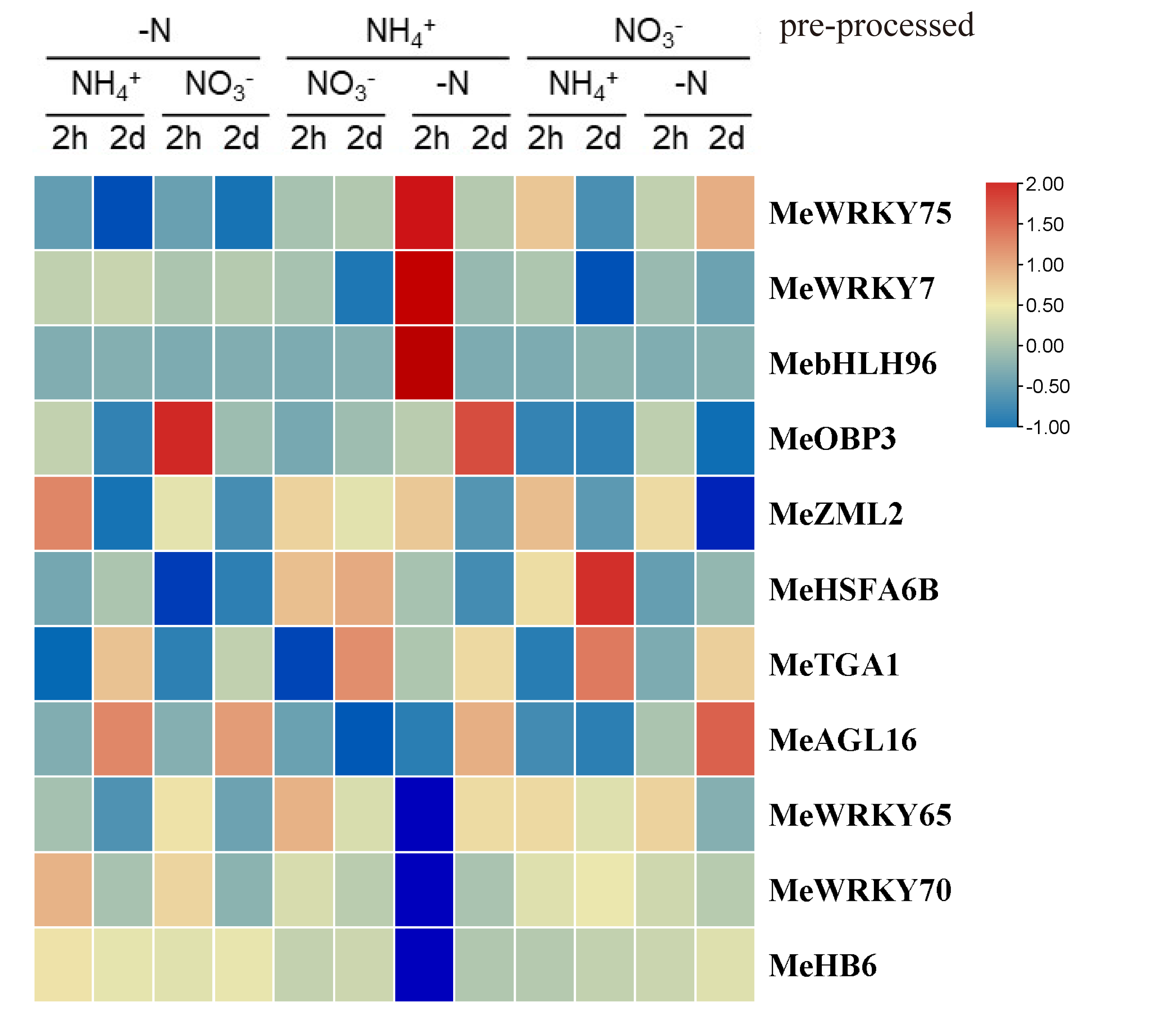

Supplement: Supplementary file 1 [file plants-14-01264-s001.zip › Supplementary Figure S7.tif]
